# Characterization of a Multiresistance Plasmid Carrying the *optrA* and *cfr* Resistance Genes From an *Enterococcus faecium* Clinical Isolate

**DOI:** 10.3389/fmicb.2018.02189

**Published:** 2018-09-11

**Authors:** Gianluca Morroni, Andrea Brenciani, Alberto Antonelli, Marco Maria D’Andrea, Vincenzo Di Pilato, Simona Fioriti, Marina Mingoia, Carla Vignaroli, Oscar Cirioni, Francesca Biavasco, Pietro E. Varaldo, Gian Maria Rossolini, Eleonora Giovanetti

**Affiliations:** ^1^Infectious Diseases Clinic, Department of Biomedical Sciences and Public Health, Polytechnic University of Marche Medical School, Ancona, Italy; ^2^Unit of Microbiology, Department of Biomedical Sciences and Public Health, Polytechnic University of Marche Medical School, Ancona, Italy; ^3^Department of Experimental and Clinical Medicine, University of Florence, Florence, Italy; ^4^Department of Medical Biotechnologies, University of Siena, Siena, Italy; ^5^Unit of Microbiology, Department of Life and Environmental Sciences, Polytechnic University of Marche, Ancona, Italy; ^6^Microbiology and Virology Unit, Florence Careggi University Hospital, Florence, Italy

**Keywords:** multiresistance plasmid, *optrA* gene, *cfr* gene, oxazolidinone resistance, *Enterococcus faecium*

## Abstract

*Enterococcus faecium* E35048, a bloodstream isolate from Italy, was the first strain where the oxazolidinone resistance gene *optrA* was detected outside China. The strain was also positive for the oxazolidinone resistance gene *cfr*. WGS analysis revealed that the two genes were linked (23.1 kb apart), being co-carried by a 41,816-bp plasmid that was named pE35048-oc. This plasmid also carried the macrolide resistance gene *erm*(B) and a backbone related to that of the well-known *Enterococcus faecalis* plasmid pRE25 (identity 96%, coverage 65%). The *optrA* gene context was original, *optrA* being part of a composite transposon, named Tn*6628*, which was integrated into the gene encoding for the ζ toxin protein (*orf19* of pRE25). The *cfr* gene was flanked by two IS*Enfa5* insertion sequences and the element was inserted into an *lnu*(E) gene. Both *optrA* and *cfr* contexts were excisable. pE35048-oc could not be transferred to enterococcal recipients by conjugation or transformation. A plasmid-cured derivative of *E. faecium* E35048 was obtained following growth at 42°C, and the complete loss of pE35048-oc was confirmed by WGS. pE35048-oc exhibited some similarity but also notable differences from pEF12-0805, a recently described enterococcal plasmid from human *E. faecium* also co-carrying *optrA* and *cfr*; conversely it was completely unrelated to other *optrA*- and *cfr*-carrying plasmids from *Staphylococcus sciuri*. The *optrA*-*cfr* linkage is a matter of concern since it could herald the possibility of a co-spread of the two genes, both involved in resistance to last resort agents such as the oxazolidinones.

## Introduction

Enterococci are members of the gut microbiota of humans and many animals, and are widespread in the environment. They are also major opportunistic pathogens, mostly causing healthcare-related infections. Among the reasons of their increasing role as nosocomial pathogens, the primary factor is their inherent ability to express and acquire resistance to several antimicrobial agents, with *Enterococcus faecium* emerging as the most therapeutically challenging species (Arias and Murray, 2012).

Oxazolidinones are among the few agents that retain activity against multiresistant strains of enterococci (Shaw and Barbachyn, 2011; Patel and Gallagher, 2015), and the emergence of resistance to these drugs is an issue of notable clinical relevance. Particularly worrisome, due to their potential for horizontal dissemination, are the oxazolidinone resistances caused by *cfr*, encoding a ribosome-modifying enzyme (Kehrenberg et al., 2005; Deshpande et al., 2015; Munita et al., 2015), and *optrA*, encoding a ribosome protection mechanism (Wang et al., 2015; Wilson, 2016; Sharkey et al., 2016). Both these genes were found to be associated with a number of different mobile genetic elements.

The *optrA* gene, in particular, was discovered in China in enterococci of human and animal origin isolated in 2005-2014 (Wang et al., 2015) where it was detected in different genetic contexts (He et al., 2016). Since then, *optrA*-positive enterococci have been reported worldwide (Mendes et al., 2016; Cavaco et al., 2017; Freitas et al., 2017; Pfaller et al., 2017a,b), including Italy, where *optrA* was found — the first report outside China — in two bloodstream isolates of *E. faecium* which were also positive for the *cfr* gene, which was not expressed (Brenciani et al., 2016b). By further investigating one of those isolates (strain E35048), we noticed that both *optrA* and *cfr* were capable of undergoing excision as minicircles (Brenciani et al., 2016b). It is worth noting that among the reported *optrA* protein variants (Morroni et al., 2017), the one detected in *E. faecium* E35048, named *optrA*_E35048_, is the most divergent, differing by 21 amino acid substitutions from the firstly described *optrA* variant (Wang et al., 2015).

The goal of the present work was to investigate the locations, genetic environments, and transferability of the *optrA* and *cfr* resistance genes detected in *E. faecium* E35048. We characterized the genetic contexts and location of *optrA* and *cfr* in *E. faecium* E35048, and found that both genes were co-carried on a plasmid of original structure, named pE35048-oc. This plasmid, which also carried the macrolide resistance gene *erm*(B), shared regions of homology with the well-characterized (Schwarz et al., 2001) and widely distributed (Rosvoll et al., 2010; Freitas et al., 2016) conjugative multiresistance enterococcal plasmid pRE25, but was unable to transfer. In pE35048-oc, the genetic context of *optrA* was different from those so far described in other *optrA*-carrying plasmids, underscoring the plasticity of these resistance regions.

## Materials and Methods

### Bacterial Strain

*optrA*- and *cfr*-positive *E. faecium* E35048 (linezolid MIC, 4 μg/ml; tedizolid MIC, 2 μg/ml) was isolated in Italy in 2015 from a blood culture (Brenciani et al., 2016b).

### WGS and Sequence Analysis

Genomic DNA was extracted using a commercial kit (Sigma-Aldrich, St. Louis, MO). WGS was carried out with the Illumina MiSeq platform (Illumina Inc., San Diego, CA, United States) by using a 2 × 300 paired end approach and a DNA library prepared using Nextera XT DNA Sample Prep Kit (Illumina, San Diego, CA, United States). *De novo* assembly was performed with SPAdes V 3.10.0 (Bankevich et al., 2012) using default parameters. Scaffolds characterized by a length ≤ 300 bp were filtered out. Raw reads were mapped to the filtered scaffolds by using bwa (Li and Durbin, 2009) to check the quality of the assembly. Tentative ordering of selected scaffolds of plasmid origin was performed by BLASTN comparisons of data from WGS to homologous plasmids, and eventually confirmed by PCR approach followed by Sanger sequencing. The ST was determined through the Center for Genomic Epidemiology^1^. Analysis of insertion sequences was carried out using ISFinder online database^2^ (Siguier et al., 2006).

### PCR Mapping Experiments

PCR mapping with outward-directed primers topo-FW (5′-GAAGCGACAAGAGCAAGTAT-3′) and optrA-RV (5′- TCTTGAACTACTGATTCTCGG-3′), and Sanger sequencing were used to close the pE35048-oc plasmid sequence.

**FIGURE 1:**
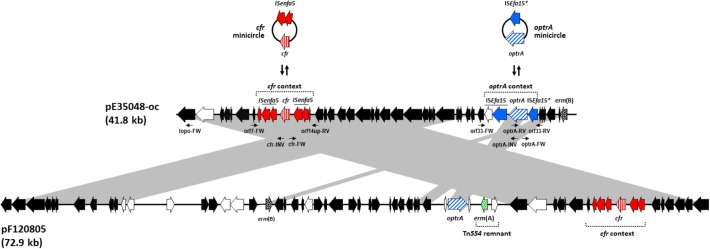
Schematic, but to scale, comparative representation of the linearized forms of plasmid pE35048-oc and plasmid pEF12-0805, both co-carrying *optrA* and *cfr* and sharing a pRE25-related backbone. ORFs are depicted as arrows pointing to the direction of transcription; those common to pRE25 are black, with *erm*(B) spotted; the *erm*(A) gene, not found in pRE25, is green spotted; the ORFs of the *optrA* and the *cfr* contexts are blue and red, respectively, with *optrA* diagonally and *cfr* vertically striped. Minicircle formation by such contexts in pE35048-oc is shown above the plasmid. Other ORFs are white. The primer pairs used are indicated by thin arrows below pE35048-oc. Gray areas between ORF maps denote > 90% DNA identity.

To investigate the excision of the *optrA* and *cfr* genetic contexts, PCR mapping and sequencing assays were performed using: (i) primer pairs targeting the regions flanking their insertion sites [orf7-FW (5′-ATTTTTCTTTTGATTTGGTA-3′) and orf14up-RV (5′-AAGTAATCTTTTTTTGTTTT-3′) for the *cfr* genetic context; and orf33-FW (5′-CTTGTTTTGGTGTTGCCCTGG-3′) and orf33-RV (5′-CCACCAAGTAAAAAAGCGG-3′) for the *optrA* genetic context]; (ii) outward-directed primer pairs designed from *cfr* and *optrA* genes [cfr-INV (5′-TTGATGACCTAATAAATGGAAGTA-3′) and cfr-FW (5′-ACCTGAGATGTATGGAGAAG-3′); optrA-INV (5′-TTTTTCCACATCCATTTCTACC-3′) and optrA-FW (5′-GAAAAATAACACAGTAAAAGGC-3′)] (**Figure 1**).

### S1-PFGE, Southern Blotting and Hybridization

Total DNA in agarose gel plugs was digested with S1 nuclease (Thermo Fisher Scientific, Milan, Italy) and separated by PFGE as previously described (Barton et al., 1995). After S1-PFGE, DNA was blotted onto positively charged nylon membrane (Ambion-Celbio, Milan, Italy) and hybridized with specific probes (Brenciani et al., 2007). *cfr* and *optrA* probes were obtained by PCR as described elsewhere (Wang et al., 2015; Brenciani et al., 2016a).

### Transformation and Conjugation Experiments

Purified plasmids extracted from *E. faecium* E35048 were transformed into the *E. faecalis* JH2-2 recipient by electrotransformation as described previously (Brenciani et al., 2016a). The transformants were selected on plates supplemented with florfenicol (10 μg/ml) or erythromycin (10 μg/ml).

In mating experiments, *E. faecium* E35048 was used as the donor. Two florfenicol-susceptible laboratory strains were used as recipients: *E. faecium* 64/3 (Werner et al., 1997), and *E. faecalis* JH2-2, both resistant to fusidic acid. Conjugal transfer was performed on a membrane filter. Transconjugants were selected on plates supplemented with florfenicol (10 μg/ml) or erythromycin (10 μg/ml) plus fusidic acid (25 μg/ml).

### Curing Assays

*Enterococcus faecium* E35048 was grown overnight in brain heart agar (BHA) at 42°C for some passages. After each passage a few colonies were picked up, and their DNA was extracted and screened for the presence of the *optrA* and *cfr* genes by PCR with specific primers (Brenciani et al., 2016b). In case of negative testing, the strain was regarded as possibly cured and subjected to WGS for confirmation.

### Nucleotide Sequence Accession Numbers

The complete nucleotide sequence of plasmid pE35048-oc has been assigned to GenBank accession no. MF580438, available under the BioProject ID PRJNA481862.

## Results and Discussion

### Genome General Features and Resistome of *E. faecium* E35048

Assembly of the raw WGS data followed by filtering of low length contigs gave a total of 172 scaffolds (range: 310-137, 266 bp; N50: 41,930 bp; L50: 18; mean coverage: 92X). *E. faecium* E35048 was assigned to ST117, a globally disseminated hospital-adapted clone (Hegstad et al., 2014; Tedim et al., 2017). Resistome analysis revealed the presence of six acquired resistance genes in addition to the previously described *optrA* and *cfr* genes: *erm*(B) (resistance to macrolides, lincosamides and group B streptogramins), *msr*(C) (resistance to macrolides and group B streptogramins), *tet*(M) (resistance to tetracycline), *aphA* and *aadE* (resistance to aminoglycosides), and *sat4* (resistance to streptothricin).

### Characterization of the *optrA*- and *cfr*-Carrying Plasmid pE35048-oc

The *optrA* and *cfr* genes were found to be linked, 23.1 kb apart in the linearized form, on the same contig, which also contained regions of high similarity (96% nucleotide identity) to the *E. faecalis* plasmid pRE25 (50 kb) (65% coverage) (Schwarz et al., 2001) (GenBank accession no. NC_008445).

PCR and Sanger sequencing using outward-directed primers targeting *orf1* and *optrA* demonstrated that the region containing *optrA* and *cfr* was part of a plasmid which was designated pE35048-oc (**Figure 1**). The plasmid was 41,816 bp in size, contained 42 open reading frames (ORFs), and had a G + C content of 35%.

S1-PFGE analysis of genomic DNA extracted from *E. faecium* E35048 showed four plasmids, ranging in size from ∼10 to ∼250 kb (data not shown). Both *optrA* and *cfr* probes hybridized with a plasmid of ∼45 kb, in agreement with sequencing data.

The characteristics of the plasmid ORFs and of their products are detailed in **Table 1**. In particular, pE35048-oc carried (i) a *repS* gene (*orf6*, corresponding to *orf6* of pRE25), encoding a theta mechanism replication protein responsible for the plasmid replication; (ii) a putative origin of replication downstream of *orf6*; and (iii) a region containing the putative minimal conjugative unit of pRE25, consisting of 15 ORFs (*orf28* to *orf14*, corresponding to *orf24* to *orf39* of pRE25) and the origin of transfer (*oriT*) found upstream of *orf28* (Schwarz et al., 2001). BLASTN analysis showed that the *oriT* nucleotide sequence was shorter in pE35048-oc (only 15 bp vs. 38 bp in pRE25). Compared to pRE25, pE35048-oc lacked (i) the region spanning from *orf41* to *orf5* (two IS*1216* elements probably involved in the rearrangement occurred during plasmid evolution); (ii) *orf10*, i.e., the chloramphenicol resistance *cat* gene; and (iii) *orf11*, another replication gene encoding a rolling-circle replication protein. In addition, compared to pRE25, pE35048-oc carried the *optrA* and *cfr* genes and their respective genetic environments.

**Table 1:** Amino acid sequence identities/similarities of putative proteins encoded by the pE35048-oc (GenBank accession no. MF580438).

					BLASTP analysis*^a^*		
ORF	Start (bp)	Stop (bp)	Size (amino Acid)	Predicted function	Most significant database match	Accession no.	% Amino acid identity (% aminacid similarity)
*orfl*	1833	1	610	DNA Topoisomerase III	Type 1 topoisomerase (plasmid) [*Enterococcus faecium*]	YP_976069.1	100 (100)
*orfl*	3,818	1,932	628	Group II intron	Group II intron reverse transcriptase/maturase [*Lactobacillales*]	WP_010718345.1	100 (100)
*^b^*Δ*orf3*	4,917	4,555	120	DNA Topoisomerase III	Topoisomerase [*Bacilli*]	WP_000108744.1	100 (100)
*orf4*	5,534	4,917	205	Resolvase	Resolvase (plasmid) [*E. faecalis*]	YP_003864109.1	99 (100)
*orf5*	5,718	5,548	56		Hypothetical protein pRE25p07 (plasmid) [*E. faecalis*]	YP_783891.1	98 (100)
*orf6*	7,557	6,067	496	Replication protein	Replication protein (plasmid) [*E. faecium*]	NP_044463.1	100 (100)
*orf7*	8,213	7,941	90	Transcriptional regulator	CopS (plasmid) [*Streptococcus pyogenes*]	YP_232751.1	100 (100)
Δ*orf8*	8,901	8,446	151	Responsible for lincomycin resistance	Lincosamide nucleotidyltransferase (plasmid) [*E. faecium*]	ARQ19308.1	99 (100)
*orf9*	9,772	8,873	299	Transposase	Transposase [*Streptococcus suis*]	AGO02197.1	100 (100)
*orf10*	10,443	9,769	224	Transposase	IS3 family transposase [*E. faecalis*]	WP_013330754.1	100 (100)
*orf11*	11,867	10,809	352	23S ribosomal RNA methyltransferase	Cfr family 23S ribosomal RNA methyltransferase [*Staphylococcus aureus*]	WP_001835153.1	100 (100)
*orf12*	13,177	12,278	299	Transposase	Transposase [*S. suis*]	AGO02197.1	100 (100)
*orf13*	13,848	13,174	224	Transposase	IS3 family transposase [*E. faecalis*]	WP_013330754.1	100 (100)
Δ*orf8*	13,921	14,061	47	Responsible for lincomycin resistance	Lincomycin resistance protein [synthetic construct]	AGT57825.1	100 (100)
*orf14*	15,391	14,531	289		Hypothetical protein [*S. suis*]	WP_079268203.1	96 (98)
*orf15*	15,822	15, 451	123		Hypothetical protein [*Enterococcus casseliflavus*]	WP_032495652.1	99 (99)
*orf16*	16,546	15,809	245		Hypothetical protein [*E. faecalis*]	WP_012858057.1	100 (100)
*orf17*	17,768	16,836	310		Hypothetical protein [*Enterococcus* sp. HMSC063D12]	WP_070544061.1	100 (100)
*orf18*	18,693	17,770	307	Membrane protein insertase	Hypothetical protein [*Enterococcus* sp. HMSC063D12]	WP_070544063.1	99 (99)
*orf19*	20,366	18,711	551	Type IV secretory pathway, VirD4 component, TraG/TraD family ATPase	Hypothetical protein [*Enterococcus*]	WP_002325630.1	100 (100)
*orf20*	20,790	20,359	143		Ypsilon (plasmid) [*E. faecalis*]	YP_003864141.1	100 (100)
*orf21*	21,346	20,795	183		Hypothetical protein [*E. faecium*]	WP_02 9485693.1	99 (99)
*orf22*	22,468	21,359	369	Amidase	Putative lytic transglycosylase (plasmid) [*E. faecalis*]	YP_003864139.1	99 (99)
*orf23*	23,842	22,490	450		Conjugal transfer protein TraF [*E. faecium*]	WP_085837474.1	98 (99)
*orf24*	25,817	23,856	653	Type IV secretory pathway, VirB4 component	TrsE (plasmid) [*E. faecalis*]	YP_003864137.1	100 (100)
*orf25*	26,457	25,828	209		Hypothetical protein [*Enterococcus*]	WP_002325627.1	99 (100)
*orf26*	26,857	26,474	127		AM21 (plasmid) [*E. faecalis*]	YP 003305365.1	100 (100)
*orf27*	27,208	26,876	110	T4SS_CagC	Hypothetical protein pRE25p25 (plasmid) [*E. faecalis*]	YP_783909.1	100 (100)
*orf28*	29,217	27,232	661	Nickase	Molybdopterin-guanine dinucleotide biosynthesis protein MobA [*E. faecalis*]	WP_025186512.1	99 (100)
*orf29*	29,509	29,808	99		Hypothetical protein pRE25p23 (plasmid) [*E. faecalis*]	YP_783907.1	100 (100)
*orf30*	29,811	30,068	85		Hypothetical protein [*Enterococcus*]	WP_021109234.1	100 (100)
*orf31*	30,927	30,430	165	Molecular chaperone DnaJ	Molecular chaperone DnaJ [*Enterococcus*]	WP_025481726.1	97 (98)
*orf32*	31,353	30,946	135		Hypothetical protein pRE25p20 (plasmid) [*E. faecalis*]	YP_783904.1	98 (99)
Δ*orf33*	32,376	31,705	223	Zeta-toxin	Toxin zeta [*E. faecium*]	WP_002300569.1	97 (98)
*orf34*	33,173	32,412	253	DNA replication protein DnaC	AAA family ATPase [*Proteiniborus ethanoligenes*]	WP_091728780.1	94 (98)
*orf35*	34,750	33,170	526	IS*Efa15* transposase	Transposase [*P. ethanoligenes*]	WP_091728892.1	70 (84)
*orf36*	36,973	35,006	655	ABC-F type ribosomal protection protein	ABC-F type ribosomal protection protein OptrA [*E. faecalis*]	WP_078122475.1	97 (98)
*orf37*	38,100	37,078	340	IS*Efa15* transposase (partial)	Transposase [*Clostridium formicaceticum*]	WP_070963420.1	64 (80)
Δ*orf33*	38,423	39,199	75	Zeta-toxin	Zeta toxin [*E. faecium*]	WP_080440976.1	100 (100)
*orf38*	38,697	38,425	90	Epsilon-antitoxin	Antidote of epsilon-zeta post-segregational killing system (plasmid) [*S. pyogenes*]	YP_232758.1	100 (100)
*orf39*	38,929	38,714	71	Omega-repressor	Transcriptional repressor (plasmid) [*S. pyogenes*]	YP_232757.1	99 (100)
*orf40*	39,917	39,021	298	ParA putative ATPase	Chromosome partitioning protein ParA [*S. suis*]	WP_0023 87620.1	100 (100)
*orf41*	40,445	40,314	43		Hypothetical protein (plasmid) [*Pediococcus acidilactici*]	WP_002321978.1	100 (100)
*orf42*	41,187	40,450	245	23S rRNA (adenine(2058)-N(6)) methyltransferase	23S rRNA (adenine(2058)-N(6))-methyltransferase Erm(B) [*S. suis*]	WP_024418925.1	99 (100)

^a^*For each ORF, only the most significant identity detected is listed. *^*b*^*Δ represents a truncated ORF.*

The *optrA* context (5,850 bp) consisted of the *optrA_E35048_* gene followed by a novel insertion sequence of the IS*21* family, named IS*Efa15*. Consistently with other members of this family (Berger and Haas, 2001), IS*Efa15* included two CDS encoding a transposase and a helper protein, and was bounded by 11-bp imperfect inverted repeats (IRL 5′-TGTTTATGATA-3′ and IRR 5′-TGTATTTGTCA-3′). A truncated copy of IS*Efa15*, named IS*Efa15^∗^*, was present also upstream of *optrA* gene. This *optrA* context was flanked by 5-bp target site duplications (5′-CTAAT-3′) suggesting its mobilization as a composite transposon, named Tn*6628* (**Figure 1**). This transposon was previously shown to form circular intermediate (3,350 bp) including *optrA* and the truncated copy of IS*Efa15* (Brenciani et al., 2016b).

The proposed role of IS*1216* in the dissemination of *optrA* among different types of enterococcal plasmids (He et al., 2016) is likely to be true also for other transposase genes. The *optrA* context was located downstream of the *erm*(B) gene (*orf15* of pRE25) and was integrated into *orf33* (*orf19* of pRE25, which encodes the ζ toxin protein of the ω-ε-ζ toxin/antitoxin system). This integration inactivates ζ toxin encoded by *orf33*, a condition that could prevent the correct partitioning of pE35048-oc and lead to the appearance of plasmid-free segregants (Magnuson, 2007).

The *cfr* context (6,098 bp) was located between *orf7* and *orf14* (*orf39* and *orf40* of pRE25) and consisted of the *cfr* gene flanked by two IS*Enfa5* elements, inserted in turn into the *lnu*(E) gene. The same genetic context of *cfr* [including the direct repeats and the *lnu*(E) gene] has been reported in China in a plasmid from a *Streptococcus suis* isolate from an apparently healthy pig (Wang et al., 2013) and in Italy in an MRSA isolated from a patient with cystic fibrosis (Antonelli et al., 2016), with *cfr* being untransferable in both instances. Very recently, the *cfr* gene, flanked by only one IS*Enfa5*, inserted upstream, has been described in a chromosomal fragment shared by three pig isolates of *Staphylococcus sciuri* (Fan et al., 2017).

PCR assays, using primer pairs targeting regions flanking the *optrA* and the *cfr* contexts (**Figure 1**), and sequencing experiments confirmed that both genes could be excised leaving one of the two flanking genes (IS*Efa15* or IS*Enfa5*, respectively) at the excision sites.

### Transferability of the *optrA* and *cfr* Genes and Curing of *E. faecium* E35048 From pE35048-oc

Repeated attempts of conjugation and transformation assays failed to demonstrate any *optrA* or *cfr* transfer from *E. faecium* E35048 to enterococcal recipients. The partial deletion of *oriT* and the lack of the rolling-circle replication protein might be responsible for the non-conjugative behavior of pE35048-oc compared to pRE25 (Schwarz et al., 2001).

An *optrA*- and *cfr*-negative isogenic strain of *E. faecium* E35048 was obtained after three passages on BHA at 42°C. It was subjected to WGS. Compared to the wild type, it disclosed complete loss of pE35048-oc.

### pE35048-oc vs. Other Plasmids Sharing Co-carriage of *optrA* and *cfr*

Since this study was started, co-location of *optrA* and *cfr* has been reported in a few additional plasmids, some from pig isolates of *S. sciuri* (Li et al., 2016; Fan et al., 2017) and one, pEF12-0805, from a human isolate of *E. faecium* (Lazaris et al., 2017). Comparison of pE35048-oc with the *S. sciuri* plasmids revealed completely unrelated backbones and *optrA* and *cfr* contexts. On the other hand, pE35048-oc was related with pEF12-0805 (accession no. KY579372.1) although with significant differences (**Figure 1**). In particular:

(i) pE35048-oc and pEF12-0805 share a pRE25-related backbone (Schwarz et al., 2001), but pEF12-0805 is much larger (72,924 bp vs. 41,816 bp) due to the presence of a larger amount of pRE25-related regions, including the pRE25 region spanning from *orf51* to *orf5* (∼12,5 kb) and a rearranged region of pRE25 containing antibiotic resistance genes *aphA*, *aadE*, and *lnu*(B) (∼13 kb). (ii) A ∼4-kb remnant of the *ermA*-carrying transposon Tn*554* (Murphy et al., 1985) is found only in pEF12-0805. (iii) The *optrA* contexts of the two plasmids are completely different, only the *optrA* gene of pE35048-oc being part of a composite transposon. The absence of insertion sequences makes it unlikely that the *optrA* gene of pEF12-0805 is excisable. Moreover, whereas in pE35048-oc the *optrA* context is found downstream of *erm*(B), the *optrA* gene of pEF12-0805 is associated with the *ermA*-carrying Tn*554* remnant. (iv) Interestingly, the *cfr* contexts of the two plasmids are the same, including some plasmid backbone flanking regions on either side (**Figure 1**), suggesting that the two plasmids might be derived from a pRE25-related common ancestor that had initially acquired the mobile *cfr* element. (v) Repeated transfer assays were unsuccessful with both plasmids. Finally, (vi) whereas we obtained a pE35048-oc-cured derivative of our *E. faecium* isolate, curing assays were unsuccessful with *E. faecium* strain F120805 (Lazaris et al., 2017).

The *E. faecium* hosts of the two plasmids belonged to different sequence types and were isolated from different sources. Strain E35048 was recovered in 2015 in Italy from a blood culture, belonged to ST117, exhibited no mutational mechanisms of oxazolidinone resistance, and was vancomycin susceptible. Strain F120805, recovered in 2013 in Ireland from feces and reported to have a linezolid MIC of 8 μg/ml, belonged to ST80, exhibited also mutational mechanisms of oxazolidinone resistance (involving both 23S rRNA and ribosomal protein L3), and was vancomycin resistant (*vanA* genotype). Although belonging to different sequence types, ST80 and ST117 were part of the same clonal group, ST78.

## Conclusion

Distinctive findings of the *optrA*- and *cfr*-carrying plasmid pE35048-oc are its relation to the well-known enterococcal plasmid pRE25, shared with plasmid pEF12-0805 (Lazaris et al., 2017); a unique *optrA* context, that has never been described before; and the fact that both the *optrA* and *cfr* contexts are capable of excising to form minicircles. This, in addition to the belonging of *E. faecium* E35048 to ST117, a globally disseminated clone recovered in many European health institutions (Hegstad et al., 2014; Tedim et al., 2017), might favor the spread of *optrA* and *cfr* in the hospital setting. Under this respect, the *in vitro* non-transferability of pE35048-oc is someway reassuring, although transfer *in vivo* cannot be ruled out. Moreover, at the hospital level, it cannot be excluded that co-carriage of *optrA* and *cfr* by the same plasmid ends up turning into co-spread, as already highlighted with pheromone-responsiveness plasmids (Francia and Clewell, 2002), and also in consideration of the very recent finding that, in enterococci, non-conjugative plasmids can be mobilized by co-resident, conjugative plasmids (Di Sante et al., 2017). Co-spread would be a cause for special concern, considering that both *optrA* and *cfr* encode resistance, through diverse mechanisms, to different antibiotics, including last resort agents such as oxazolidinones.

## Author Contributions

AB, PV, and EG designed the study and wrote the paper. FB, OC, and GR have contributed to critical reading of the manuscript. GM, AA, MD, VD, SF, MM, CV, and SF did the laboratory work.

## Conflict of Interest Statement

The authors declare that the research was conducted in the absence of any commercial or financial relationships that could be construed as a potential conflict of interest.

## Footnotes

1 https://cge.cbs.dtu.dk/services/MLST/

2 https://isfinder.biotoul.fr/

## References

[B1] Antonelli A. D’Andrea M. M. Galano A. Borchi B. Brenciani A. Vaggelli G. (2016). Linezolid-resistant *cfr*-positive MRSA, Italy. *J. Antimicrob. Chemother.* 71 2349–2351. 10.1093/jac/dkw108 27073268

[B2] Arias C. A. Murray B. E. (2012). The rise of the *Enterococcus*: beyond vancomycin resistance. *Nat. Rev. Microbiol.* 10 266–278. 10.1038/nrmicro2761 22421879 PMC3621121

[B3] Bankevich A. Nurk S. Antipov D. Gurevich A. A. Dvorkin M. Kulikov A. S. (2012). SPAdes: a new genome assembly algorithm and its applications to single-cell sequencing. *J. Comput. Biol.* 19 455–477. 10.1089/cmb.2012.0021 22506599 PMC3342519

[B4] Barton B. M. Harding G. P. Zuccarelli A. J. (1995). A general method for detecting and sizing large plasmids. *Anal. Biochem.* 226 235–240. 10.1006/abio.1995.1220 7793624

[B5] Berger B. Haas D. (2001). Transposase and cointegrase: specialized transposition proteins of the bacterial insertion sequence IS*21* and related elements. *Cell. Mol. Life Sci.* 58 403–419. 10.1007/PL00000866 11315188 PMC11337337

[B6] Brenciani A. Bacciaglia A. Vecchi M. Vitali L. A. Varaldo P. E. Giovanetti E. (2007). Genetic elements carrying *erm*(B) in *Streptococcus pyogenes* and association with *tet*(M) tetracycline resistance gene. *Antimicrob. Agents Chemother.* 51 1209–1216. 10.1128/AAC.01484-06 17261630 PMC1855496

[B7] Brenciani A. Morroni G. Pollini S. Tiberi E. Mingoia M. Varaldo P. E. (2016a). Characterization of novel conjugative multiresistance plasmids carrying *cfr* from linezolid-resistant *Staphylococcus epidermidis* clinical isolates from Italy. *J. Antimicrob. Chemother.* 71 307–313. 10.1093/jac/dkv341 26472766

[B8] Brenciani A. Morroni G. Vincenzi C. Manso E. Mingoia M. Giovanetti E. (2016b). Detection in Italy of two clinical *Enterococcus faecium* isolates carrying both the oxazolidinone and phenicol resistance gene *optrA* and a silent multiresistance gene *cfr*. *J. Antimicrob. Chemother.* 71 1118–1119. 10.1093/jac/dkv438 26702919

[B9] Cavaco L. M. Bernal J. F. Zankari E. Léon M. Hendriksen R. S. Perez-Gutierrez E. (2017). Detection of linezolid resistance due to the *optrA* gene in *Enterococcus faecalis* from poultry meat from the American continent (Colombia). *J. Antimicrob. Chemother.* 72 678–683. 10.1093/jac/dkw490 27999039

[B10] Deshpande L. M. Ashcraft D. S. Kahn H. P. Pankey G. Jones R. N. Farrell D. J. (2015). Detection of a new *cfr*-like gene, *cfr*(B), in *Enterococcus faecium* isolates recovered from human specimens in the United States as part of the SENTRY antimicrobial surveillance program. *Antimicrob. Agents Chemother.* 59 6256–6261. 10.1128/AAC.01473-15 26248384 PMC4576063

[B11] Di Sante L. Morroni G. Brenciani A. Vignaroli C. Antonelli A. D’Andrea M. M. (2017). pHTβ-promoted mobilization of non-conjugative resistance plasmids from *Enterococcus faecium* to *Enterococcus faecalis*. *J. Antimicrob. Chemother.* 72 2447–2453. 10.1093/jac/dkx197 28645197

[B12] Fan R. Lia D. Feßler A. T. Wu C. Schwarz S. Wang Y. (2017). Distribution of *optrA* and *cfr* in florfenicol-resistant *Staphylococcus sciuri* of pig origin. *Vet. Microbiol.* 210 43–48. 10.1016/j.vetmic.2017.07.030 29103695

[B13] Francia M. V. Clewell D. B. (2002). Transfer origins in the conjugative *Enterococcus faecalis* plasmids pAD1 and pAM373: identification of the pAD1 nic site, a specific relaxase and a possible TraG-like protein. *Mol. Microbiol.* 45 375–395. 10.1046/j.1365-2958.2002.03007.x 12123451

[B14] Freitas A. R. Elghaieb H. León-Sampedro R. Abbassi M. S. Novais C. Coque T. M. (2017). Detection of *optrA* in the African continent (Tunisia) within a mosaic *Enterococcus faecalis* plasmid from urban wastewaters. *J. Antimicrob. Chemother.* 72 3245–3251. 10.1093/jac/dkx321 29029072

[B15] Freitas A. R. Tedim A. P. Francia M. V. Jensen L. B. Novais C. Peixe L. (2016). Multilevel population genetic analysis of *vanA* and *vanB Enterococcus faecium* causing nosocomial outbreaks in 27 countries (1986-2012). *J. Antimicrob. Chemother.* 71 3351–3366. 10.1093/jac/dkw312 27530756

[B16] He T. Shen Y. Schwarz S. Cai J. Lv Y. Li J. (2016). Genetic environment of the transferable oxazolidinone/phenicol resistance gene *optrA* in *Enterococcus faecalis* isolates of human and animal origin. *J. Antimicrob. Chemother.* 71 1466–1473. 10.1093/jac/dkw016 26903276

[B17] Hegstad K. Longva J. Å. Hide R. Aasnæs B. Lunde T. M. Simonsen G. S. (2014). Cluster of linezolid-resistant *Enterococcus faecium* ST117 in Norwegian hospitals. *Scand. J. Infect. Dis.* 46 712–715. 10.3109/00365548.2014.923107 25134650

[B18] Kehrenberg C. Schwarz S. Jacobsen L. Hansen L. H. Vester B. (2005). A new mechanism for chloramphenicol, florfenicol and clindamycin resistance: methylation of 23S ribosomal RNA at A2503. *Mol. Microbiol.* 57 1064–1073. 10.1111/j.1365-2958.2005.04754.x 16091044

[B19] Lazaris A. Coleman D. C. Kearns A. M. Pichon B. Kinnevey P. M. Earls M. R. (2017). Novel multiresistance *cfr* plasmids in linezolid-resistant methicillin-resistant *Staphylococcus epidermidis* and vancomycin-resistant *Enterococcus faecium* (VRE) from a hospital outbreak: co-location of *cfr* and *optrA* in VRE. *J. Antimicrob. Chemother.* 72 3252–3257. 10.1093/jac/dkx292 28961986

[B20] Li D. Wang Y. Schwarz S. Cai J. Fan R. Li J. (2016). Co-location of the oxazolidinone resistance genes *optrA* and *cfr* on a multiresistance plasmid from *Staphylococcus sciuri*. *J. Antimicrob. Chemother.* 71 1474–1478. 10.1093/jac/dkw040 26953332

[B21] Li H. Durbin R. (2009). Fast and accurate short read alignment with burrows-wheeler transform. *Bioinformatics.* 25 1754–1760. 10.1093/bioinformatics/btp324 19451168 PMC2705234

[B22] Magnuson R. D. (2007). Hypothetical functions of toxin-antitoxin systems. *J. Bacteriol.* 189 6089–6092. 10.1128/JB.00958-07 17616596 PMC1951896

[B23] Mendes R. E. Hogan P. A. Jones R. N. Sader H. S. Flamm R. K. (2016). Surveillance for linezolid resistance via the Zyvox® annual appraisal of potency and spectrum (ZAAPS) programme (2014): evolving resistance mechanisms with stable susceptibility rates. *J. Antimicrob. Chemother.* 71 1860–1865. 10.1093/jac/dkw052 27013481

[B24] Morroni G. Brenciani A. Simoni S. Vignaroli C. Mingoia M. Giovanetti E. (2017). Commentary: nationwide surveillance of novel oxazolidinone resistance gene *optrA* in *Enterococcus* isolates in China from 2004 to 2014. *Front. Microbiol.* 8:1631. 10.3389/fmicb.2017.01631 28883817 PMC5573801

[B25] Munita J. M. Bayer A. S. Arias C. A. (2015). Evolving resistance among gram-positive pathogens. *Clin. Infect. Dis.* 61 48–57. 10.1093/cid/civ523 26316558 PMC4551095

[B26] Murphy E. Huwyler L. de Freire Bastos Mdo C. (1985). Transposon Tn554: complete nucleotide sequence and isolation of transposition-defective and antibiotic-sensitive mutants. *EMBO J.* 4 3357–3365. 3004956 10.1002/j.1460-2075.1985.tb04089.x PMC554666

[B27] Patel R. Gallagher J. C. (2015). Vancomycin-resistant enterococcal bacteremia pharmacotherapy. *Ann. Pharmacother.* 49 69–85. 10.1177/1060028014556879 25352037

[B28] Pfaller M. A. Mendes R. E. Streit J. M. Hogan P. A. Flamm R. K. (2017a). Five-year summary of in vitro activity and resistance mechanisms of linezolid against clinically important gram-positive cocci in the United States from the LEADER surveillance program (2011 to 2015). *Antimicrob. Agents. Chemother.* 61:e00609-17. 10.1128/AAC.00609-17 28483950 PMC5487612

[B29] Pfaller M. A. Mendes R. E. Streit J. M. Hogan P. A. Flamm R. K. (2017b). ZAAPS Program results for 2015: an activity and spectrum analysis of linezolid using clinical isolates from medical centres in 32 countries. *J. Antimicrob. Chemother.* 72 3093–3099. 10.1093/jac/dkx251 28961701

[B30] Rosvoll T. C. S. Pedersen T. Sletvold H. Johnsen P. J. Sollid J. E. Simonsen G. S. (2010). PCR-based plasmid typing in *Enterococcus faecium* strains reveals widely distributed pRE25-, pRUM-, pIP501- and pHTβ-related replicons associated with glycopeptide resistance and stabilizing toxin-antitoxin systems. *FEMS Immunol. Med. Microbiol.* 58 254–268. 10.1111/j.1574-695X.2009.00633.x 20015231

[B31] Schwarz F. V. Perreten V. Teuber M. (2001). Sequence of the 50-kb conjugative multiresistance plasmid pRE25 from *Enterococcus faecalis* RE25. *Plasmid* 46 170–177. 10.1006/plas.2001.1544 11735367

[B32] Sharkey L. K. R. Edwards T. A. O’Neill A. J. (2016). ABC-F proteins mediate antibiotic resistance through ribosomal protection. *mBio* 7:e01975. 10.1128/mBio.01975-15 27006457 PMC4807367

[B33] Shaw K. J. Barbachyn M. R. (2011). The oxazolidinones: past, present, and future. *Ann. N. Y. Acad. Sci.* 1241 48–70. 10.1111/j.1749-6632.2011.06330.x 22191526

[B34] Siguier P. Perochon J. Lestrade L. Mahillon J. Chandler M. (2006). ISfinder: the reference centre for bacterial insertion sequences. *Nucleic Acids Res.* 34 D32–D36. 10.1093/nar/gkj014 16381877 PMC1347377

[B35] Tedim A. P. Lanza V. F. Manrique M. Pareja E. Ruiz-Garbajosa P. Cantón R. (2017). Complete genome sequences of isolates of *Enterococcus faecium* sequence type 117, a globally disseminated multidrug-resistant clone. *Genome Announc.* 5:e01553-16. 10.1128/genomeA.01553-16 28360174 PMC5374248

[B36] Wang Y. Li D. Song L. Liu Y. He T. Liu H. (2013). First report of the multiresistance gene *cfr* in *Streptococcus suis*. *Antimicrob. Agents Chemother.* 57 4061–4063. 10.1128/AAC.00713-13 23733472 PMC3719703

[B37] Wang Y. Lv Y. Cai J. Schwarz S. Cui L. Hu Z. (2015). A novel gene, *optrA*, that confers transferable resistance to oxazolidinones and phenicols and its presence in *Enterococcus faecalis* and *Enterococcus faecium* of human and animal origin. *J. Antimicrob. Chemother.* 70 2182–2190. 10.1093/jac/dkv116 25977397

[B38] Werner G. Klare I. Witte W. (1997). Arrangement of the *vanA* gene cluster in enterococci of different ecological origin. *FEMS Microbiol. Lett.* 155 55–61. 10.1111/j.1574-6968.1997.tb12685.x 9345764

[B39] Wilson D. N. (2016). The ABC of ribosome-related antibiotic resistance. *mBio* 7:e00598-16. 10.1128/mBio.00598-16 27143393 PMC4959660

